# Prevalence of high blood pressure and its associated factors among students in Shenyang, China: A cross-sectional study

**DOI:** 10.1097/MD.0000000000035536

**Published:** 2023-10-20

**Authors:** Dan Zhang, Baijun Sun, Xiaodan Yi, Nan Dong, Guifang Gong, Wenbo Yu, Lianying Guo

**Affiliations:** a Department of School Health, Shenyang Center for Disease Control and Prevention, Shenyang, China; b Department of Nutrition and Food Hygiene, School of Public Health, Shenyang Medical College, Shenyang, China.

**Keywords:** BMI, high blood pressure, prevalence, sleep duration, students

## Abstract

There is growing evidence that the prevalence of high blood pressure is increasing, and it may have serious consequences. However, research on the prevalence and influencing factors of high blood pressure among primary and secondary school students is still relatively scarce. This study aims to investigate the prevalence and influencing factors of high blood pressure among primary and secondary school students in Shenyang, in order to provide scientific evidence for the prevention and management of this disease. From April to May 2020, 4892 students aged 7 to 17 years were selected as the survey subjects, and on-site physical measurements and questionnaire surveys were conducted. The prevalence of high blood pressure was described. Restricted cubic spline was used to analyze the dose-response relationship between sleep duration, BMI and the risk of high blood pressure. Logistic regression was used to analyze the risk factors. Multiplicative and additive models were used to analyze the interaction between sleep duration and BMI. The results showed that the overall prevalence of high blood pressure among students aged 7 to 17 years in Shenyang was 9.9%, with a higher prevalence in females than males (12.1% vs 7.9%) and in urban areas than suburban areas (11.8% vs 7.7%). The prevalence was lowest in students with normal weight (8.3%) and highest in those who were obese (12.5%). The prevalence fluctuated to some extent among different age groups, but overall, it increased with age, with the lowest prevalence in primary school students (7.0%), 11.4% in mild school students, and the highest prevalence of 14.3% in high school students. Multivariable analysis showed that the risk of high blood pressure in female students was 1.90 times higher than that in male students (95% CI: 1.54–2.35), and the risk in suburban areas was 0.65 times lower than that in urban areas (95% CI: 0.52–0.81). Students with a BMI ≥ 21 kg/m^2^ had a 1.58 times higher risk than those with a BMI < 21 kg/m^2^(95% CI: 1.28–1.96), while those with a sleep time ≥ 8 hours had a 0.80 times lower risk than those with a sleep time < 8 hours (95% CI: 0.65–0.99). Exercise can significantly reduce the risk of high blood pressure, while using electronic devices for more than 0.5 hours significantly increases the risk of high blood pressure. BMI and sleep duration have no interaction effect on the risk of high blood pressure. To reduce the prevalence of high blood pressure, students should reduce the use of electronic devices, ensure adequate exercise, maintain a reasonable weight, and ensure sufficient sleep.

## 1. Introduction

According to the Global Burden of Cardiovascular Diseases and Risk Factors study (1990–2019), cardiovascular diseases are the leading cause of death worldwide, and hypertension is one of the most important risk factors for cardiovascular diseases.^[1]^ Hypertension has become a major public health issue worldwide, with serious impacts on people’s health.

With the changes in lifestyle, worsening environmental pollution, and increasing academic pressure, the prevalence of high blood pressure among students has also significantly increased.^[2–5]^ Previous “tracking” blood pressure studies have shown that hypertension in adults can often be predicted by blood pressure in childhood, and children with high blood pressure are at increased risk for hypertension and metabolic syndrome later in life.^[6,7]^ A meta-analysis also indicated a close correlation between childhood blood pressure and blood pressure in later years.^[8]^Recent studies have shown that high blood pressure in young people significantly increases the risk of death from cardiovascular diseases such as stroke and coronary heart disease, and can cause damage to the urinary and endocrine systems.^[9–11]^ The study by Dionne et al^[12]^ indicated that identifying and treating children and adolescents with high blood pressure may help prevent the occurrence of long-term cardiovascular diseases.

In summary, although high blood pressure in children and adolescents may be asymptomatic, it will seriously affect their future health. Therefore, the problem of high blood pressure in children and adolescents should be taken seriously. Chen and Wang^[8]^ emphasized the importance of early intervention, but currently, research on the prevalence of high blood pressure and its influencing factors among children and adolescent students is relatively scarce, especially in China where there are few related reports.

Previous epidemiological surveys have shown that the risk factors for hypertension in adults are high in Shenyang, China,^[13–15]^ but there has been no investigation into the prevalence and influencing factors of high blood pressure among primary and secondary school students in this area. This study investigated the current prevalence and influencing factors of high blood pressure among 7 to 17 years students in Shenyang, China, filling this gap.

## 2. Materials and methods

### 2.1. Study design and settings

This study was a cross-sectional survey conducted from April to May 2020, using a multi-stage stratified cluster sampling method. One district (Heping District) was randomly selected from the 5 urban districts in Shenyang, and one suburban county (Xinmin City) was randomly selected from the 8 suburban counties as the survey site. Two primary schools, 2 middle schools, and 2 high schools were randomly selected in each survey site, and one vocational school was selected if available, resulting in a total of 13 schools, including 4 primary schools, 4 mild schools, 4 high schools, and one vocational school. In each grade of the selected schools, 2 to 3 classes were randomly selected (ensuring that there were more than 100 students in each grade) as the survey subjects.

Shenyang is the capital of Liaoning Province, the largest city in Northeast China, and a multi-ethnic city with a large population and a large number of primary and secondary school students. Therefore, the survey results also have good guiding significance for other regions.

### 2.2. Data collection

#### 2.2.1. Blood pressure measurement.

Blood pressure measurement was performed on-site at the school by professional doctors from the testing team using an upper arm electronic sphygmomanometer (Welcon, XW-700) certified by international standard protocols (European Society of Hypertension-ESH, British Hypertension Society-BHS, Association for the Advancement of Medical Instrumentation-AAMI). The subjects were advised not to engage in vigorous physical activity within 1 to 2 hours before the first blood pressure measurement. Prior to the measurement, the subjects were instructed to sit quietly for 10 to 15 minutes, stabilize their emotions. When measuring blood pressure, the subjects were seated with the right arm naturally extended and kept at the same level as the heart. The measurement is repeated after 1 to 2 minutes, and the average of 2 readings is recorded. If the difference between the 2 readings of systolic or diastolic blood pressure is more than 5 mm Hg, the measurement is repeated, and the average of 3 readings is recorded. For blood pressure retesting, the subjects must rest for an additional 10 to 15 minutes before the measurement can be taken. The measurement results were recorded on-site into the Liaoning Province Student Common Diseases and Health Influencing Factors Monitoring Information System.

#### 2.2.2. Height measurement.

Height measurement was performed on-site at the school by professional personnel of the testing team in groups of 2 (one person testing, one person recording). A mechanical height meter (SuHong, ZG-8053) was used. According to the instructions, before use, calibrated the zero point and placed a “standard steel ruler” on the bottom plate of the height meter to check the height meter scale and whether the height of the red line on the benchmark plate is 10.0 cm, with an error not exceeding 0.1 cm. At the same time, checked whether the column was against the wall, whether it was vertical, whether the connection was tight, whether there was any shaking, and whether the parts were loose. When measuring, the examinee stood barefoot with their back to the column, standing on the bottom plate of the height meter, with their torso naturally straight, head straight, and eyes looked straight ahead (the upper edge of the earphone and the lowest point of the eye socket were horizontal); the upper limbs were naturally drooped, and both legs were straight; the heels were together, and the toes were about 60° apart; the heels, sacrum, and scapulae contacted the column, forming a “three-point-one-line” standing posture. The detection personnel used one hand to slide the horizontal pressure plate down along the column to the top of the examinee’s head. When reading the measurement, the detection personnel’s eyes were at the same height as the plane of the horizontal pressure plate. The value was recorded in the system in centimeters, accurate to 1 decimal place.

#### 2.2.3. Weight measurement.

Weight measurement was performed on-site at the school by professional personnel of the testing team using an electronic weighting scale (SuHong, RCS-200). Before use, the working status, accuracy of the weighting scale were checked according to the instructions. First, the electronic weighing scale was placed on a flat surface and the power switch was turned on. Waited for a few seconds until the digital display showed “0.00,” indicating that the scale had been initialized to zero. Next, the calibration weight (1000 g) was placed on the scale’s platform and waited for a while until the digital display stabilized and showed the weight of the calibration weight. If the weight displayed on the digital display did not match the actual weight of the calibration weight, we used the adjustment knob to calibrate it. When measuring, the examinee wears short sleeves and shorts, barefoot, and stands naturally in the center of the weighing plate, keeping the body stable. After the value displayed on the screen were stable, the detection personnel recorded the displayed value. The value was recorded in the system in kilograms, accurate to 1 decimal place.

#### 2.2.4. Questionnaire Survey.

Students were organized to fill out the questionnaire survey on the WeChat official account (considering that students in grades 1 to 3 of primary school have insufficient reading comprehension level, no questionnaire survey is arranged for them). The survey content mainly included the basic information of the participants, dietary behavior, exercise behavior, electronic device usage, and sleep duration. The specific items were listed in Table 1. The survey questionnaire results were directly uploaded to the Liaoning Province Student Common Disease and Health Impact Factor Monitoring Information System.

**Table 1:** Baseline characteristics of all participants.

Characteristic	Overall, N = 4892 (100%)*	Normal blood pressure, N = 4408 (90%)*	High blood pressure, N = 484 (10%)*	*P* value†
Age (yr)	12.0 (9.0, 15.0)	12.0 (9.0, 14.0)	13.0 (11.0, 15.0)	<.001
Sex				
Male	2602 (53.2%)	2396 (54.4%)	206 (42.6%)	<.001
Female	2290 (46.8%)	2012 (45.6%)	278 (57.4%)	
Ethnicity				
Han	3995 (81.7%)	3607 (81.8%)	388 (80.2%)	.400
Others	897 (18.3%)	801 (18.2%)	96 (19.8%)	
Region				
Urban	2600 (53.1%)	2293 (52.0%)	307 (63.4%)	<.001
Suburban	2292 (46.9%)	2115 (48.0%)	177 (36.6%)	
Educational stage				
Primary school	2403 (49.1%)	2257 (51.2%)	169 (34.9%)	<.001
Mild school	1274 (26.0%)	1154 (26.2%)	149 (30.8%)	
High school	1215 (24.8%)	997 (22.6%)	166 (34.3%)	
BMI (kg/m^2^)	20.4 (17.7, 24.1)	20.3 (17.6, 23.8)	21.9 (18.9, 27.3)	<.001
SBP (mm Hg)	105.0 (96.0, 112.0)	103.0 (95.0, 110.0)	120.0 (110.0, 127.3)	<.001
DBP (mm Hg)	69.0 (63.0, 74.0)	68.0 (62.0, 72.0)	80.0 (80.0, 85.0)	<.001

BMI = body mass index, DBP = diastolic blood pressure, SBP = systolic blood pressure.

* Median (IQR); n (%).

† Wilcoxon rank sum test; Pearson’s Chi-squared test.

### 2.3. Related diagnosis and definitions

Diagnosis of high blood pressure in students was based on the Chinese national health industry standard: Threshold for screening elevated blood pressure in children and adolescents (WS/T 610-2018). Children and adolescents of both genders aged 7 to 17 years with systolic blood pressure and/or diastolic blood pressure equal to or greater than the 95th percentile of blood pressure for the same gender, age, and height were classified as having high blood pressure.

Body mass index (BMI) was calculated as weight (kg) divided by height (m) squared. Diagnosis for underweight in students was based on the Chinese national health industry standard: Screening standard for malnutrition of school-age children and adolescents (WS/T 456-2014), while diagnosis for overweight and obesity was based on the Chinese national health industry standard: Screening for overweight and obesity among school-age children and adolescent (WS/T 586-2018).

### 2.4. Quality control

The detection personnel who participated in the survey used uniform detection methods and equipment. The detection team was composed of 1 team leader, at least one professional with a national practicing physician qualification or a nurse qualification certificate, and a professional in the field of school health. All detection personnel received uniform training and were proficient in detection methods. They could only be on the job after passing the assessment.

During the daily detection process, a random sample of 5% of the subjects were selected for height and weight retests to verify the detection error (height exceeding 0.5 cm, weight exceeding 0.1 kg is considered an error). If the error rate was greater than 5%, the team leader should promptly convene a meeting to study the reasons and improvement measures, and conduct retests and corrections for indicators that exceed the allowable error range. If the error rate is greater than 10%, all detection data for the day were invalid and must be retested.

### 2.5. Statistical analysis

EpiData version 3.1 (EpiData Association, Odense, Denmark) software was used to establish the database, and parallel double data entry was performed. R version 4.2.3 (R Foundation for Statistical Computing, Vienna, Austria) software was used for statistical analysis. Metric data were described using mean ± standard deviation or median (quartile), and count data were described using frequency (percentage). The restricted cubic spline (RCS) function is a useful tool to describe the dose-response relationships between continuous exposure and outcomes. In this study, the RCS function was implemented in the “rms” package (Version 6.7) in R software (https://cran.r-project.org/web/packages/rms/). To prevent overfitting, the package recommends using 3 to 4 knots and visually selecting the narrowest 95% confidence interval as a clue for determining the cut points. We used an RCS model with 3 knots to conduct dose-response analysis of the relationship between sleep duration, BMI, and the risk of high blood pressure.

The logistic regression analysis was used to analyze the risk factors. First, a univariable logistic regression analysis was performed, and the variables with *P* < .1 were selected as independent variables, and high blood pressure was selected as the dependent variable for multivariable analysis. The odds ratio (OR) and its 95% confidence interval (CI) were given. The interaction between sleep duration and BMI on high blood pressure was analyzed by multiplication and addition. The relative excess risk of interaction (RERI), attributable proportion of interaction (AP), and synergy index (SI) were used to evaluate the additive interaction. If the confidence intervals of RERI and AP include 0, and the confidence interval of SI includes 1, there is no interaction. The threshold for significant differences was set at *P* < .05.

### 2.6. Ethics approval and consent to participate

This study received review and approval from the Ethics Committee of the Shenyang Center for Disease Control and Prevention, with the ethics code of SYCDC-2020-001. Written informed consent was obtained from the legal guardians of the participants for their participation in this study.

## 3. Result

### 3.1. Basic characteristics of participants

As shown in Table 1, a total of 4892 students aged 7 to 17 years were analyzed in this study, with a median age of 12.0 years (IQR: 9.0, 15.0). Among them, boys accounted for 53.2%, slightly higher than girls. The ethnic group was mainly Han, accounting for 81.7%. The urban area accounted for 54.4%, slightly higher than the suburban area. Primary school students accounted for 49.6%, while mild and high school students accounted for a similar proportion. The median BMI was 20.4 kg/m^2^ (IQR: 17.70, 24.1). The median systolic and diastolic blood pressures were 105.0 mm Hg (IQR: 6.0, 112.0) and 69.0 mm Hg (IQR: 63.0, 74.0), respectively. Except for ethnicity, other variables showed statistically significant differences between the normal blood pressure group and the high blood pressure group (*P* < .05).

### 3.2. Prevalence of high blood pressure

**Figure 1.**
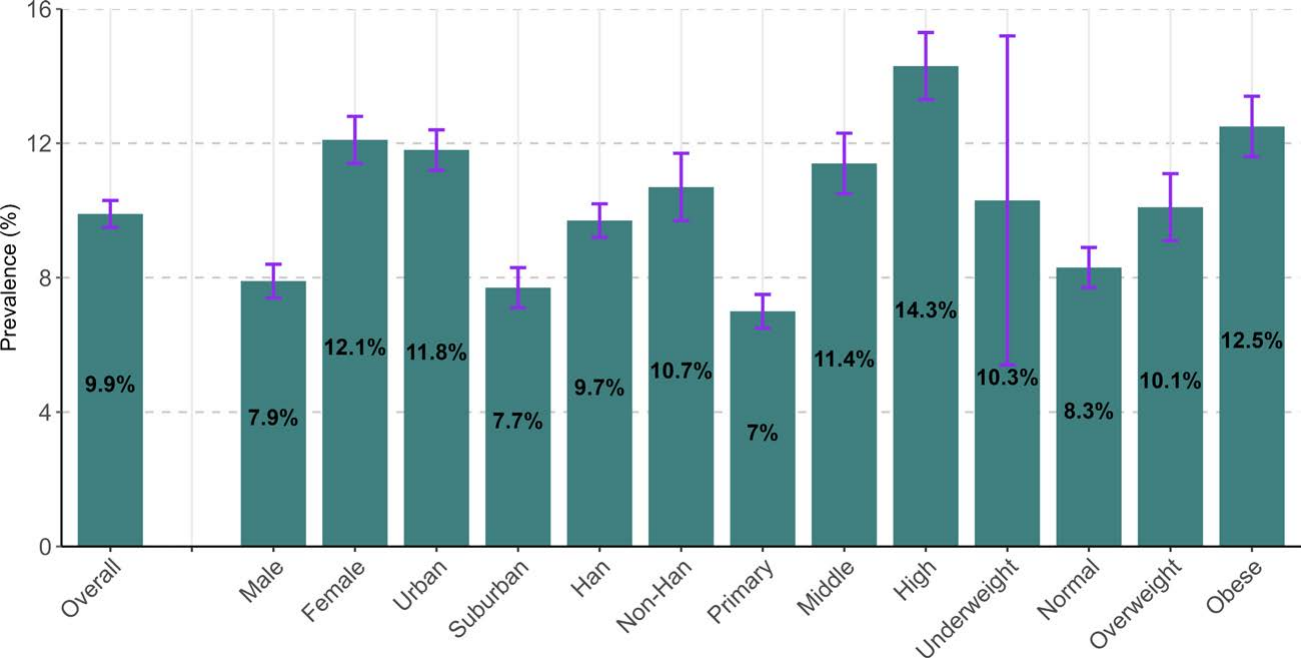
Prevalence of high blood pressure among students aged 7–17 years.

Figure 1 (Table S1, Supplemental Digital Content, http://links.lww.com/MD/K253) showed that among the 4892 students surveyed, 484 had high blood pressure, with an overall prevalence of 9.9% (95% CI: 9.5–10.3). The prevalence among female students was significantly higher at 12.1% (95% CI: 11.4–12.8) compared to male students at 7.9% (95% CI: 7.4–8.4), and in urban areas (11.8%, 95% CI: 11.2–12.4) was significantly higher than that in suburban areas (7.7%, 95% CI: 7.1–8.3).The prevalence among Han students was slightly lower at 9.7% (95% CI: 9.2–10.2) compared to non-Han students at 10.7% (95% CI: 9.7–11.7).The prevalence increased gradually with educational stage, from 7.0% (95% CI: 6.5–7.5) in primary school to 11.4% (95% CI: 10.5–12.3)in middle school and 14.3% (95% CI: 13.3–15.3) in high school. The prevalence among students with normal weight was the lowest (8.3%, 95% CI: 7.7–8.9), while the prevalence among underweight and overweight students was similar, at 10.3% (95% CI: 5.4–15.2) and 10.1% (95% CI: 9.1–15.2), respectively. The prevalence was highest among obese students, at 12.5% (95% CI: 11.6–13.4).

### 3.3. Prevalence of high blood pressure at different ages

**Figure 2.**
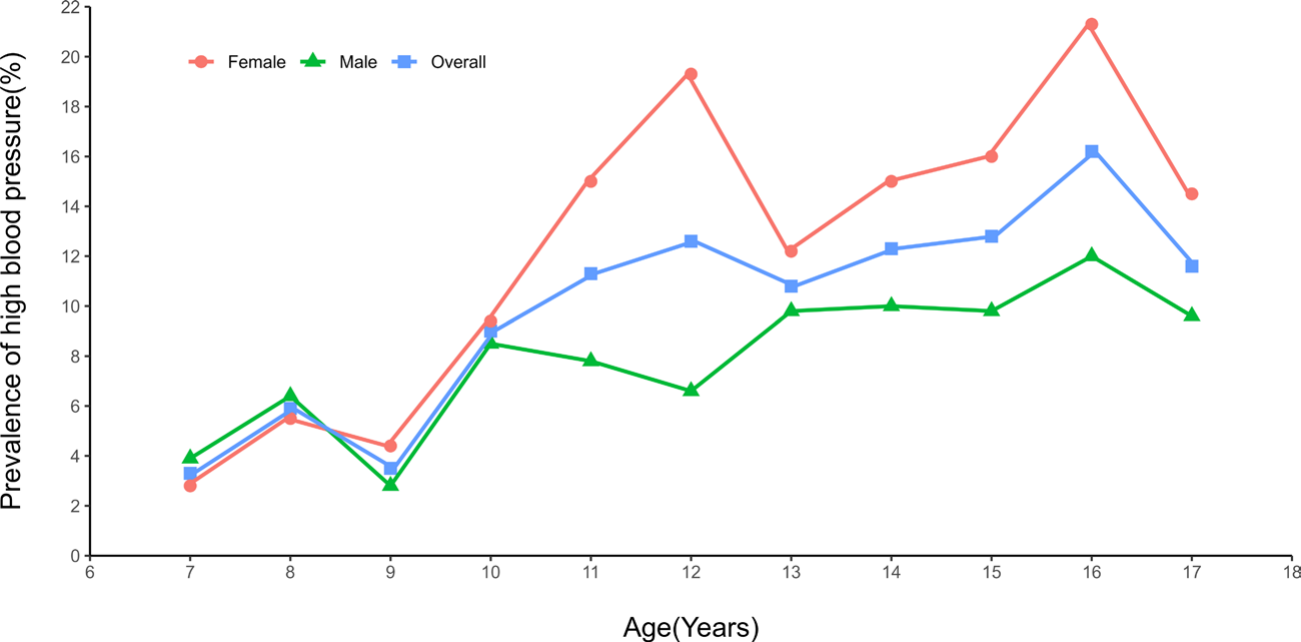
Prevalence of high blood pressure at different ages.

As shown in Figure 2 (Table S2, Supplemental Digital Content, http://links.lww.com/MD/K254), the prevalence of high blood pressure fluctuated to some extent at different ages, but overall, it showed an increasing trend with age. Particularly, the prevalence was higher in females, with a faster and more volatile increase, while males had a lower prevalence with a relatively stable and slower increase.

### 3.4. Dose-response between BMI, sleep duration and the risk of high blood pressure

**Figure 3.**
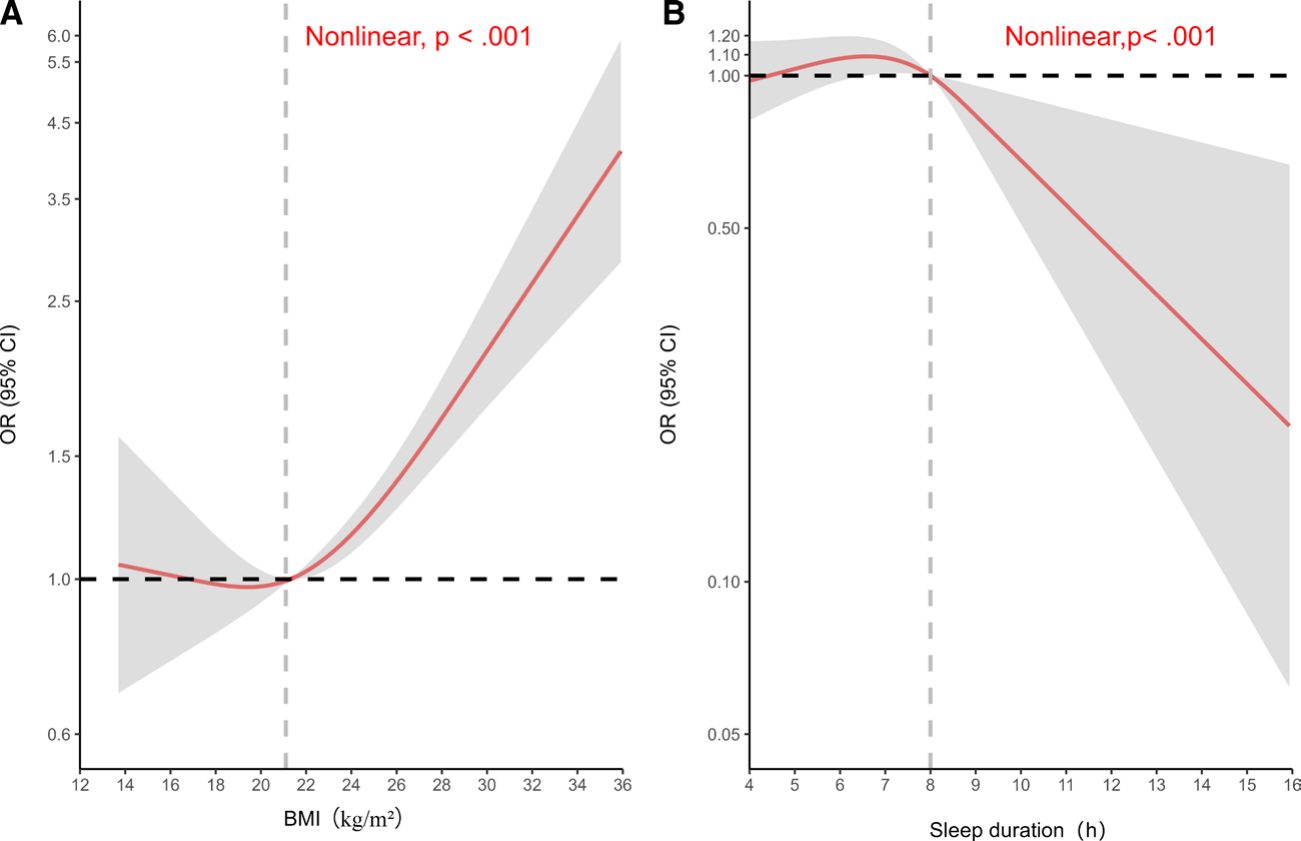
Dose-response between BMI, sleep duration and the risk of high blood pressure. *The y-axis is logarithmically scaled. (A) Dose-response between BMI and the risk of high blood pressure. adjusted for age, sex, region, physical activity, hours of mobile electronic devices use, sleep duration, smoke, drink, fruit, vegetable, breakfast. (B) Dose-response between sleep duration and the risk of high blood pressure. adjusted for age, sex, region, BMI, physical activity, hours of mobile electronic devices use, smoke, drink, fruit, vegetable, breakfast. BMI = body mass index, CI = confidence interval, OR = odds ratio.

The restricted cubic spline analysis (Fig. 3) showed that BMI, sleep duration, and the risk of high blood pressure all had a non-linear relationship. When BMI was less than 21 kg/m^2^, BMI had little impact on the risk of high blood pressure. However, when BMI was greater than 21 kg/m^2^, the risk of high blood pressure increased rapidly with increasing BMI. Sleeping less than 8 hours per day increased the risk of high blood pressure, while sleeping more than 8 hours per day significantly reduced the risk of high blood pressure.

### 3.5. Risk factors for high blood pressure

**Figure 4.**
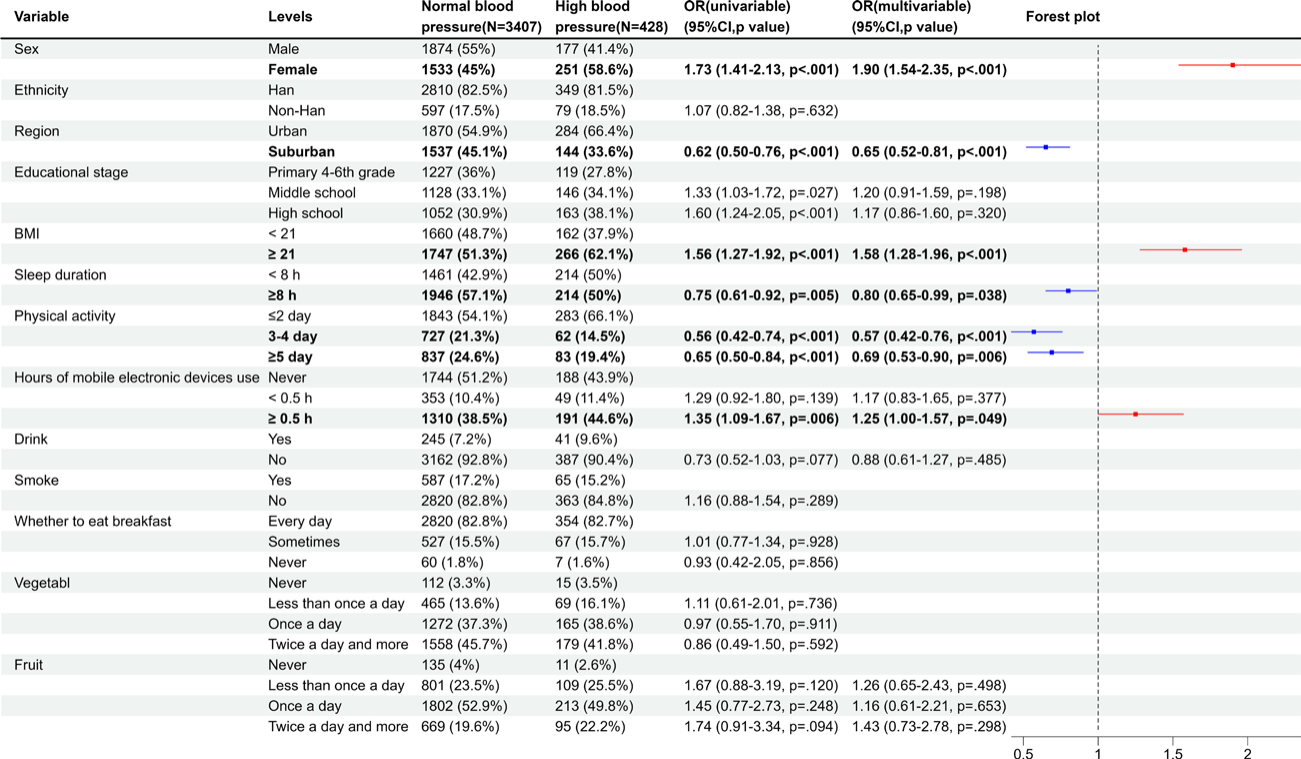
Forest plot of logistic regression results of risk factors for high blood pressure. (1) Physical activity: How many days in the past week have you achieved at least 60 minutes of moderate to high-intensity exercise per day? Moderate-intensity exercise refers to physical activity that makes you breathe harder or increases your heart rate, such as running, basketball, football, swimming, and heavy lifting. (2) Drink: Have you consumed a whole glass of alcohol? (equivalent to one can of beer, one small bowl of liquor, one glass of wine or yellow wine). (3) Smoke: In the past 30 days, have you smoked? CI = confidence interval, OR = odds ratio.

Due to the lack of participation of students in grades 1 to 3 in the questionnaire survey, a total of 3835 students participated in the analysis of risk factors, among which 3407 students had normal blood pressure and 428 students had high blood pressure. The differences in characteristics between participants and non-participants were shown in Table 2. Univariable analysis showed that being female, being in a higher grade, having a BMI ≥ 21 kg/m^2^, and using electronic devices for ≥ 0.5 hours were risk factors for high blood pressure, while living in suburban areas, having a sleep duration ≥ 8 hours, and engaging in moderate-to-high intensity physical activity for 3 or more days per week were protective factors. Multivariable analysis showed that the risk of high blood pressure in female students was 1.90 times higher than that in male students (95% CI: 1.54–2.35), and the risk in suburban areas was 0.65 times lower than that in urban areas (95% CI: 0.52–0.81). Students with a BMI ≥ 21 kg/m^2^ had a 1.58 times higher risk of high blood pressure than those with a BMI < 21 kg/m^2^ (95% CI: 1.28–1.96), while those with a sleep time ≥ 8 hours had a 0.80 times lower risk than those with a sleep time < 8 hours (95% CI: 0.65–0.99). Compared with students who engaged in high-intensity exercise 2 days or less per week, those who engaged in high–intensity exercise 3 to 4 days per week had a 0.57 times lower risk (95% CI: 0.42–0.76), and those who engaged in high–intensity exercise 5 days or more per week had a 0.69 times lower risk (95% CI: 0.53–0.90). The risk of high blood pressure in students who used electronic devices for ≥ 0.5 hours per day was 1.25 times higher than that in those who did not use electronic devices (95% CI: 1.00–1.57) (Fig. 4).

**Table 2:** Characteristics of participants involved in the analysis of factors associated with high blood pressure and non-participants.

Characteristic	Overall, N = 4892 (100%)*	Participants, N = 3835 (78%)*	Non-participants, N = 1057 (22%)*	*P* value†
Age (yr)	12.0 (9.00, 15.0)	13.0 (11.00, 15.0)	8.0 (7.00, 8.0)	<.001
High blood pressure				
No	4408 (90.1%)	3407 (88.8%)	1011 (94.7%)	<.001
Yes	484 (9.9%)	428 (11.2%)	56 (5.3%)	
Sex				
Male	2602 (53.2%)	2051 (53.5%)	551 (52.1%)	.8
Female	2290 (46.8%)	1784 (46.5%)	506 (47.9%)	
Ethnicity				
Han	3995 (81.7%)	3159 (82.4%)	836 (79.1%)	.009
Others	897 (18.3%)	676 (17.6%)	221 (20.9%)	
Region				
Urban	2600 (53.1%)	2154 (56.2%)	446 (42.2%)	<.001
Suburban	2292 (46.9%)	1681 (43.8%)	611 (57.8%)	
Educational stage				
Primary school	2403 (49.1%)	1346 (35.1%)	1057 (100.0%)	<.001
Mild school	1274 (26.0%)	1274 (33.2%)	0 (0.0%)	
High school	1215 (24.8%)	1215 (31.7%)	0 (0.0%)	
BMI (kg/m^2^)	20.4 (17.70, 24.1)	21.3 (18.60, 25.2)	17.4 (15.80, 20.4)	<.001
SBP (mm Hg)	105.0 (96.00, 112.0)	108.0 (100.00, 116.0)	93.0 (89.00, 98.0)	<.001
DBP (mm Hg)	69.0 (63.00, 74.0)	70.0 (65.00, 76.0)	63.0 (60.00, 70.0)	<.001

BMI = body mass index, DBP = diastolic blood pressure, SBP = systolic blood pressure.

* Median (IQR); n (%).

† Wilcoxon rank sum test; Pearson’s Chi-squared test.

### 3.6. Interaction between BMI and sleep duration

The results of the interaction analysis (Table 3) showed that, after adjusting for age, sex, region, physical activity, and hours of mobile electronic devices use, compared with the group with BMI < 21 kg/m^2^ and sleep duration ≥ 8 hours, the risk of high blood pressure in the group with BMI ≥ 21 kg/m^2^ and sleep duration ≥ 8 hours was 1.69 times (95% CI: 1.23–2.31); the risk in the group with BMI < 21 kg/m^2^ and sleep duration < 8 hours was 1.03 times (95% CI: 0.72–1.47); and the risk in the group with BMI ≥ 21 kg/m^2^ and sleep duration < 8 hours was 1.55 times (95% CI: 1.11–2.17). There was no multiplicative interaction between the two factors (OR = 0.89, 95% CI: 0.58–1.37). The indices reflecting additive interaction, including RERI and AP, included 0 in the confidence interval, while SI included 1, indicating no additive interaction between the two factors. The BMI stratification analysis showed that sleep duration was not associated with the risk of high blood pressure, whether in the subgroup with BMI < 21 kg/m^2^ or the subgroup with BMI ≥ 21 kg/m^2^. However, the sleep duration stratification analysis showed that BMI ≥ 21 kg/m^2^ significantly increased the risk of high blood pressure, whether in the subgroup with sleep duration ≥ 8 hours or the subgroup with sleep duration < 8 hours.

**Table 3:** Interaction of BMI and sleep duration on high blood pressure.

Indicator	Sleep duration ≥ 8 hours	Sleep duration < 8 hours	Effect of sleep duration within the strata of BMI
	OR [95% CI]	OR [95% CI]	OR [95% CI]
BMI < 21 kg/m^2^	1.00 [Ref]	1.03 [0.72, 1.47]	1.03 [0.72, 1.47]
BMI ≥ 21 kg/m^2^	1.69 [1.23, 2.31]	1.55 [1.11, 2.17]	0.92 [0.69, 1.23]
Effect of BMI within the strata of sleep duration	1.69 [1.24, 2.31]	1.52 [1.13, 2.03]	
Multiplicative scale	0.89 [0.58, 1.37]		
RERI	−0.16 [−0.73, 0.40]		
AP	−0.11 [−0.47, 0.26]		
SI	0.77 [0.34, 1.72]		

Adjusted for age, sex, region, physical activity, hours of mobile electronic devices use.

AP = attributable proportion, BMI = body mass index, CI = confidence interval, OR = odds ratios, RERI = relative excess risk of interaction, SI = synergy index.

### 3.7. Sensitivity analysis

The BMI was reclassified into 4 categories (underweight, normal, overweight, and obese), and sleep duration was divided into 3 categories (<7 hours, 7–9 hours, and ≥ 9 hours). A new logistic regression analysis was conducted using these categories. The results (Table S3, Supplemental Digital Content, http://links.lww.com/MD/K255) did not show any significant changes in the trends, indicating the robustness of the findings.

## 4. Discussion

Currently, there are not many reports on the prevalence of high blood pressure among Chinese students. Chen et al^[16]^ reported a prevalence of high blood pressure of 9.92% among students aged 6 to 17 years in Suzhou. Li et al^[17]^ reported a prevalence of 12.26% of high blood pressure among students under 18 years old in Lanzhou. Liu et al^[18]^ reported a prevalence of high blood pressure of 16.4% among students aged 7 to 17 in Sichuan Province. This study showed that the prevalence of high blood pressure among school students age 7 to 17 in Shenyang was 9.9%, which showed an upward trend compared to the national prevalence of 6.9% 10 years ago,^[19]^ but was relatively low compared to other regions. We also looked up the prevalence of high blood pressure among children and adolescents in other countries. Nagata et al^[20]^ analyzed cross-sectional data from the United States from 2018 to 2020 to understand the differences in blood pressure among American adolescents before and during the COVID-19 pandemic. The results showed that compared to before the COVID-19 pandemic, the proportion of adolescents with high blood pressure during the pandemic increased from 3.4% to 6.4%. A Spanish study including 2957 male and female students (aged 5–18 years) reported a high blood pressure prevalence of 6.6% using the 2016 European Society of Hypertension (referred to as “2016 ESH”) guidelines as the diagnostic criteria.^[21]^ A Canadian study investigating the blood pressure of 7387 children aged 6 to 18 years found a high blood pressure prevalence of 4.5% using the 2017 American Academy of Pediatrics guidelines as the diagnostic criteria. From the above reports, it can be seen that the situation of high blood pressure among children and adolescents in Shenyang and even in China is very serious, and the situation of high blood pressure among children and adolescents globally is also concerning and should be given more attention.

Regionally, we found that the prevalence of high blood pressure in urban areas was significantly higher than in suburban areas, which may be related to the higher consumption of fried and high-sugar foods leading to a higher probability of obesity in urban areas,^[22]^ greater academic pressure in urban areas relative to suburbs,^[23]^ and relatively less greenery and more severe air pollution in cities.^[24]^ In terms of gender and age, the prevalence of high blood pressure showed a rapid upward trend after the onset of puberty, with greater fluctuations, especially among females, which is consistent with the results of Ye et al^[25]^ We believe that this is related to the significant differences in hormone levels between childhood and puberty.^[26,27]^ This phenomenon is more pronounced in females, possibly related to earlier onset of puberty in females relative to males,^[28]^ and estrogen secretion.^[29]^ Moreover, compared to males, females are relatively quiet and have sedentary habits that are prone to high blood pressure.^[30]^

Multiple studies have shown that obesity and overweight are important risk factors for high blood pressure,^[14,31,32]^ and a cross-sectional study of adults in the northeastern region of China showed a gradually increasing linear relationship between weight and blood pressure.^[33]^ The results of this survey showed that the prevalence of high blood pressure in students with normal weight was the lowest, while the prevalence of high blood pressure in students with low weight, overweight, or obesity was significantly increased. Restricted cubic spline analysis showed a non-linear relationship between BMI and the risk of high blood pressure, with a significant increase in the risk of high blood pressure when BMI ≥ 21 kg/m^2^. This indicates that weight is closely related to blood pressure, and the relationship between low weight and blood pressure still needs further research to prove, while overweight or obesity can significantly increase blood pressure. Some studies have suggested that the reason for the prevalence in blood pressure with high BMI may be related to a high insulin state, which activates the sympathetic nervous system, leading to increased renal reabsorption of sodium, increased vascular volume, vascular constriction, ultimately leading to hypertension.^[34]^ Other studies have suggested that excessive or abnormal distribution of adipose tissue in obese individuals can affect the renin-angiotensin-aldosterone system, leading to an increase in blood pressure.^[35]^

Due to increased academic pressure, and the emergence of some unhealthy habits, insufficient sleep has become a common problem among primary and secondary school students.^[36–38]^ Studies have shown that insufficient sleep can affect blood pressure,^[39,40]^ and this study indicated that insufficient sleep was also a risk factor for high blood pressure in students. Restricted cubic spline analysis showed that sleep duration of less than 8 hours was a risk factor for high blood pressure. Research has shown that insufficient sleep can affect central nervous system regulation,^[41]^ hemodynamics changes,^[42]^ hormone secretion,^[43]^ and disruption of biological rhythms,^[44,45]^ leading to pathological changes in blood pressure.^[46]^ In addition, a cross-sectional study by Dashti et al^[47]^ showed that short sleep duration leading to increased total energy and fat intake also has an impact on blood pressure.

As insufficient sleep and BMI are both important risk factors for high blood pressure, and they interact with each other. Researches have shown that obesity caused by high BMI can lead to breathing pauses during sleep, which can interrupt sleep and reduce sleep duration, leading to insufficient sleep.^[48,49]^ The study by Young T et al^[50]^ showed that for every 10% increase in BMI, the risk of moderate to severe breathing pauses increased by 6 times. Conversely, insufficient sleep and its resulting disruption of circadian rhythms can affect appetite hormones, leptin, energy consumption, and lead to high BMI and obesity.^[51,52]^ These 2 risk factors interact with each other and promote each other, so this analysis conducted an interaction study on both factors. However, the results of this analysis showed that there was no interaction between insufficient sleep and BMI on the risk of high blood pressure, whether in the multiplied interaction or added interaction. The reason may be that insufficient sleep and BMI do not actually have an interaction effect on the risk of high blood pressure, or it may be because primary and secondary school students still have relatively long sleep times, and there are not many severely obese students, resulting in the lack of interaction between insufficient sleep and BMI on blood pressure.

Although the mechanism is not yet fully understood, multiple studies have shown that exercise is beneficial in reducing hypertension in adults,^[53,54]^ and the recent American hypertension treatment guidelines strongly recommend exercise as a treatment option.^[55]^ However, there is limited research on the effect of exercise on high blood pressure in primary and secondary school students. The results of this survey showed that active exercise can also significantly reduce the risk of high blood pressure in students. This study also found that prolonged use of electronic products on a daily basis is a risk factor for high blood pressure, and it may be related to sympathetic nervous system stimulation and cortisol imbalance.^[56]^ Our results suggested that students should engage in as much exercise as possible on a daily basis and reduce sedentary behavior or prolonged use of electronic devices.

### 4.1. Limitations

Firstly, this study is a cross-sectional study, so our research cannot describe the changes and growth trends of the problem. Secondly, we did not statistically control for students’ learning and life stress, which led to incomplete control of confounding factors.

## 5. Conclusion

Our study indicated that the current prevalence of high blood pressure among Chinese primary and secondary school students is not optimistic, and we need to take active action. This includes reducing sedentary time and the use of electronic devices among students, increasing their exercise time and intensity, ensuring reasonable weight, and adequate sleep time.

## Acknowledgments

The authors would like to express their gratitude to all participants for their cooperation and to all staff for their support.

## Author contributions

**Conceptualization:** Dan Zhang, Baijun Sun, Xiaodan Yi, Lianying Guo.

**Data curation:** Dan Zhang.

**Formal analysis:** Dan Zhang, Lianying Guo.

**Investigation:** Dan Zhang, Xiaodan Yi, Nan Dong, Guifang Gong, Wenbo Yu.

**Project administration:** Dan Zhang.

**Software:** Lianying Guo.

**Supervision:** Baijun Sun.

**Writing – original draft:** Dan Zhang, Lianying Guo.

**Writing – review & editing:** Dan Zhang, Lianying Guo.

## Supplementary Material






