# Supplementary material for: Prevalence of high blood pressure and its associated factors among students in Shenyang, China: A cross-sectional study
Source: Medicine (Baltimore). 2023 Oct 20;102(42):e35536. doi: 10.1097/MD.0000000000035536 (PMC10589542; doi:10.1097/MD.0000000000035536)
Supplement: Supplementary file 1 [file medi-102-e35536-s001.docx]

Supplementary Table 1 Prevalence of high blood pressure among students aged 7-17 years

| **Characteristic** | **Overall**,  N = 4,892 (100%)^1^ | **Normal blood pressure**,  N = 4,408 (90%)^1^ | **High blood pressure**,  N = 484 (9.9%)^1^ | ***p*-value**^2^ |
| --- | --- | --- | --- | --- |
| Sex |  |  |  | <.001 |
| Male | 2,602 (100.0%) | 2,396 (92.1%) | 206 (7.9%) |  |
| Female | 2,290 (100.0%) | 2,012 (87.9%) | 278 (12.1%) |  |
| Region |  |  |  | <.001 |
| Urban | 2,600 (100.0%) | 2,293 (88.2%) | 307 (11.8%) |  |
| Suburban | 2,292 (100.0%) | 2,115 (92.3%) | 177 (7.7%) |  |
| Ethnicity |  |  |  | .400 |
| Han | 3,995 (100.0%) | 3,607 (90.3%) | 388 (9.7%) |  |
| Others | 897 (100.0%) | 801 (89.3%) | 96 (10.7%) |  |
| Educational stage |  |  |  | <.001 |
| Primary school | 2,426 (100.0%) | 2,257 (93.0%) | 169 (7.0%) |  |
| Mild school | 1,303 (100.0%) | 1,154 (88.6%) | 149 (11.4%) |  |
| High school | 1,163 (100.0%) | 997 (85.7%) | 166 (14.3%) |  |
| BMI |  |  |  | <.001 |
| Underweight | 39 (100.0%) | 35 (89.7%) | 4 (10.3%) |  |
| Normal weight | 2,472 (100.0%) | 2,267 (91.7%) | 205 (8.3%) |  |
| Overweight | 949 (100.0%) | 853 (89.9%) | 96 (10.1%) |  |
| Obesity | 1,432 (100.0%) | 1,253 (87.5%) | 179 (12.5%) |  |
| ^1^n (%) | | | | |
| ^2^Pearson's Chi-squared test; Fisher's exact test | | | | |
