# Supplementary material for: Prevalence of high blood pressure and its associated factors among students in Shenyang, China: A cross-sectional study
Source: Medicine (Baltimore). 2023 Oct 20;102(42):e35536. doi: 10.1097/MD.0000000000035536 (PMC10589542; doi:10.1097/MD.0000000000035536)
Supplement: Supplementary file 2 [file medi-102-e35536-s002.docx]

| Supplementary Table 2 Prevalence of high blood pressure at different ages | | | | | | |
| --- | --- | --- | --- | --- | --- | --- |
|  | **Overall** | | **Male** | | **Female** | |
| Age (years) | Normal blood pressure,  N = 4,408^1^ | High blood pressure,  N = 484^1^ | Normal blood pressure,  N = 2,396^1^ | High blood pressure,  N = 206^1^ | Normal blood pressure,  N = 2,012^1^ | High blood pressure,  N = 278^1^ |
| 7 | 434 (96.7%) | 15 (3.3%) | 223 (96.1%) | 9 (3.9%) | 211 (97.2%) | 6 (2.8%) |
| 8 | 459 (94.1%) | 29 (5.9%) | 235 (93.6%) | 16 (6.4%) | 224 (94.5%) | 13 (5.5%) |
| 9 | 436 (96.5%) | 16 (3.5%) | 239 (97.2%) | 7 (2.8%) | 197 (95.6%) | 9 (4.4%) |
| 10 | 395 (91.0%) | 39 (9.0%) | 193 (91.5%) | 18 (8.5%) | 202 (90.6%) | 21 (9.4%) |
| 11 | 423 (88.7%) | 54 (11.3%) | 224 (92.2%) | 19 (7.8%) | 199 (85.0%) | 35 (15.0%) |
| 12 | 396 (87.4%) | 57 (12.6%) | 225 (93.4%) | 16 (6.6%) | 171 (80.7%) | 41 (19.3%) |
| 13 | 428 (89.2%) | 52 (10.8%) | 248 (90.2%) | 27 (9.8%) | 180 (87.8%) | 25 (12.2%) |
| 14 | 358 (87.7%) | 50 (12.3%) | 199 (90.0%) | 22 (10.0%) | 159 (85.0%) | 28 (15.0%) |
| 15 | 429 (87.2%) | 63 (12.8%) | 229 (90.2%) | 25 (9.8%) | 200 (84.0%) | 38 (16.0%) |
| 16 | 384 (83.8%) | 74 (16.2%) | 221 (88.0%) | 30 (12.0%) | 163 (78.7%) | 44 (21.3%) |
| 17 | 266 (88.4%) | 35 (11.6%) | 160 (90.4%) | 17 (9.6%) | 106 (85.5%) | 18 (14.5%) |
| ^1^n (%) | | | | | | |
