# Supplementary material for: Prevalence of high blood pressure and its associated factors among students in Shenyang, China: A cross-sectional study
Source: Medicine (Baltimore). 2023 Oct 20;102(42):e35536. doi: 10.1097/MD.0000000000035536 (PMC10589542; doi:10.1097/MD.0000000000035536)
Supplement: Supplementary file 3 [file medi-102-e35536-s003.docx]

| Supplementary Table 3 Logistic regression results of risk factors for high blood pressure in sensitivity analysis | | | | | |
| --- | --- | --- | --- | --- | --- |
| Variable | levels | Normal blood pressure  (N=3407) | High blood pressure  (N=428) | OR (univariable) | OR (multivariable) |
| Sex | Male | 1874 (55%) | 177 (41.4%) |  |  |
|  | Female | 1533 (45%) | 251 (58.6%) | 1.73 (1.41-2.13, p<.001) | 1.89 (1.53-2.34, p<.001) |
| Ethnicity | Han | 2810 (82.5%) | 349 (81.5%) |  |  |
|  | Non-Han | 597 (17.5%) | 79 (18.5%) | 1.07 (0.82-1.38, p=.632) |  |
| Region | Urban | 1870 (54.9%) | 284 (66.4%) |  |  |
|  | Suburban | 1537 (45.1%) | 144 (33.6%) | 0.62 (0.50-0.76, p<.001) | 0.66 (0.52-0.82, p<.001) |
| Educational stage | Primary 4-6th grade | 1227 (36%) | 119 (27.8%) |  |  |
|  | Middle school | 1128 (33.1%) | 146 (34.1%) | 1.33 (1.03-1.72, p=.027) | 1.28 (0.97-1.70, p=.077) |
|  | High school | 1052 (30.9%) | 163 (38.1%) | 1.60 (1.24-2.05, p<.001) | 1.37 (1.01-1.85, p=.043) |
| BMI | Normal | 1800 (52.8%) | 180 (42.1%) |  |  |
|  | Overweight | 653 (19.2%) | 83 (19.4%) | 1.27 (0.97-1.67, p=.087) | 1.38 (1.04-1.83, p=.024) |
|  | Obese | 922 (27.1%) | 161 (37.6%) | 1.75 (1.39-2.19, p<.001) | 2.02 (1.60-2.55, p<.001) |
|  | Underweight | 32 (0.9%) | 4 (0.9%) | 1.25 (0.44-3.57, p=.677) | 1.25 (0.43-3.63, p=.678) |
| Sleep duration | < 7 h | 987 (29%) | 146 (34.1%) |  |  |
|  | 7-9 h | 1563 (45.9%) | 204 (47.7%) | 0.88 (0.70-1.11, p=.280) | 0.90 (0.71-1.14, p=.370) |
|  | ≥9 h | 857 (25.2%) | 78 (18.2%) | 0.62 (0.46-0.82, p=.001) | 0.70 (0.52-0.95, p=.022) |
| Physical activity^a^ | ≤2 day | 1843 (54.1%) | 283 (66.1%) |  |  |
|  | 3-4 day | 727 (21.3%) | 62 (14.5%) | 0.56 (0.42-0.74, p<.001) | 0.57 (0.43-0.77, p<.001) |
|  | ≥5 day | 837 (24.6%) | 83 (19.4%) | 0.65 (0.50-0.84, p<.001) | 0.69 (0.53-0.91, p=.007) |
| Hours of mobile electronic devices use | Never | 1744 (51.2%) | 188 (43.9%) |  |  |
|  | < 0.5 h | 353 (10.4%) | 49 (11.4%) | 1.29 (0.92-1.80, p=.139) | 1.18 (0.84-1.66, p=.343) |
|  | ≥ 0.5 h | 1310 (38.5%) | 191 (44.6%) | 1.35 (1.09-1.67, p=.006) | 1.25 (1.00-1.57, p=.052) |
| Drink^b^ | Yes | 245 (7.2%) | 41 (9.6%) |  |  |
|  | No | 3162 (92.8%) | 387 (90.4%) | 0.73 (0.52-1.03, p=.077) | 0.87 (0.60-1.26, p=.458) |
| Smoke^c^ | Yes | 587 (17.2%) | 65 (15.2%) |  |  |
|  | No | 2820 (82.8%) | 363 (84.8%) | 1.16 (0.88-1.54, p=.289) |  |
| Whether to eat breakfast | Every day | 2820 (82.8%) | 354 (82.7%) |  |  |
|  | Sometimes | 527 (15.5%) | 67 (15.7%) | 1.01 (0.77-1.34, p=.928) |  |
|  | Never | 60 (1.8%) | 7 (1.6%) | 0.93 (0.42-2.05, p=.856) |  |
| Vegetable | Never | 112 (3.3%) | 15 (3.5%) |  |  |
|  | Less than once a day | 465 (13.6%) | 69 (16.1%) | 1.11 (0.61-2.01, p=.736) |  |
|  | Once a day | 1272 (37.3%) | 165 (38.6%) | 0.97 (0.55-1.70, p=.911) |  |
|  | Twice a day and more | 1558 (45.7%) | 179 (41.8%) | 0.86 (0.49-1.50, p=.592) |  |
| Fruit | Never | 135 (4%) | 11 (2.6%) |  |  |
|  | Less than once a day | 801 (23.5%) | 109 (25.5%) | 1.67 (0.88-3.19, p=.120) | 1.30 (0.67-2.52, p=.435) |
|  | Once a day | 1802 (52.9%) | 213 (49.8%) | 1.45 (0.77-2.73, p=.248) | 1.20 (0.63-2.29, p=.578) |
|  | Twice a day and more | 669 (19.6%) | 95 (22.2%) | 1.74 (0.91-3.34, p=.094) | 1.48 (0.76-2.89, p=.252) |

a:How many days in the past week have you achieved at least 60 minutes of moderate to high-intensity exercise per day? Moderate-intensity exercise refers to physical activity that makes you breathe harder or increases your heart rate, such as running, basketball, football, swimming, and heavy lifting.

b:Have you consumed a whole glass of alcohol? (equivalent to one can of beer, one small bowl of liquor, one glass of wine or yellow wine)

c:In the past 30 days, have you smoked?
